# Influence of Activator Na_2_O Concentration on Residual Strengths of Alkali-Activated Slag Mortar upon Exposure to Elevated Temperatures

**DOI:** 10.3390/ma11081296

**Published:** 2018-07-27

**Authors:** Tai Thanh Tran, Hyug-Moon Kwon

**Affiliations:** Department of Civil Engineering, Yeungnam University, Gyeongsan, Gyeongbuk 712-749, Korea; thanhtaivlxd@gmail.com

**Keywords:** alkali-activated slag, elevated temperatures, Na_2_O concentration, residual strength, brittleness, melting

## Abstract

The mechanical strength variation of ambient cured Alkali-activated mortar (AAS) upon exposure to elevated temperatures from 200 to 1200 °C was studied in this article. Slag was activated by the combination of sodium silicate liquid (Na_2_SiO_3_) and sodium hydroxide (NaOH) with different Na_2_O concentrations of 4%, 6%, 8%, and 10% by slag weight. Mechanical properties comprising compressive strength, flexural strength, and tensile strength before and after exposure were measured. Thermogravimetric analysis (Thermogravimetric analysis (TGA) and Derivative thermogravimetric (DTG)), X-ray diffraction (XRD), scanning electron microscope (SEM), and energy-dispersive X-ray spectroscopy (EDS) were also used for strength alteration explanation. The results indicated that Na_2_O concentration influence on strength variation of AAS mortar was observed clearly at temperature range from ambient temperature to 200 °C. The melting alteration of AAS mortar after exposed to 1200 °C was highly dependent on concentrations of Na_2_O.

## 1. Introduction

Blast furnace slag is a by-product that is formed by rapidly cooling the slag liquid from the furnace in cast iron manufacture [1]. For a long time, blast furnace slag is known as a mineral admixture that can be used for partial replacement of Portland cement in blended cement or concrete [1]. In recent decades, non-Portland cement binder, named as alkali activated slag (AAS), which is synthesized by mixing blast furnace slag with alkali hydroxide, carbonate, or silicate attracted a great deal of attention of many scientists due to its high strength, durability, and low environmental impact [2,3]. The main product of AAS is a low crystalline hydrated calcium silicate, like a C-S-H gel type with a low CaO/SiO_2_ ratio [4].

The activation process of slag is highly dependent on the physical-chemical properties of blast furnace slag, the nature and dosage of activator and the curing condition [5,6]. When compared with sodium carbonate or sodium hydroxide activator, slag activated by sodium silicate liquid possessed the highest compressive strength [5]. The activation products are predominantly composed of sodium and calcium aluminosilicate hydrates (C-A-S-H and N-A-S-H), as well as some hydrotalcite-like products when using sodium hydroxide or sodium silicate as activator [7,8,9,10]. Furthermore, urban and industrial glass waste was investigated to be used as a potential alkaline activator for blast furnace slag [11,12,13]. AAS binders was observed exhibit some advantages such as earlier and higher mechanical strengths, lower heat of hydration when compared with original Portland cement (OPC) and concretes [14]. However, slag mortar activated with sodium silicate liquid was reported to have a higher drying shrinkage and to be more brittle than ordinary Portland cement (OPC) mortar [15]. Previous studies indicated that none or little amount of Ca(OH)_2_ was found in AAS system [16]. Consequently, AAS is expected to exhibit stronger resistance to extremely aggressive environments, such as chemical solution or high temperature exposure [17,18,19,20,21].

In recent years, many publications investigated the thermal behavior of various AAS material when exposed to elevated temperatures. Zuda, L. et al. [22,23,24] studied the alteration of sodium silicate powder activated slag mortar using quartz sand [22,23] and electrical porcelain [24] as fine aggregate subjected to high temperatures up to 1200 °C. The results presented a decrease in residual compressive strength of exposed mortar and obtained the lowest value of approximately 20% at 800 °C. However, the residual compressive strength greatly increased from 800 to 1200 °C due to sintering phenomena between binder matrix and aggregate. At 1200 °C, the remaining strength attained about 87% of unexposed mortar strength in the case of using quartz sand as fine aggregate and a doubling of original mortar strength when using electrical porcelain. Guerrieri, M. et al. [25] studied the effect of high temperatures up to 1200 °C on properties of AAS concrete activated by powdered sodium metasilicate and hydrated lime. The study showed that the residual strength of specimens was approximately 76%, 73%, 46% and 10% of unexposed specimen strength when exposed to 200 °C, 400 °C, 600 °C, and 800 °C, respectively. Moreover, the fire performance of AAS mortar cured in two different regimes (ambient and heat curing condition) with exposure temperatures from 200 to 800 °C was investigated by Türker, H.T. et al. [26]. The results illustrated that the strength of ambient curing specimens at 200 °C increased approximately 20% when compared to reference specimens, while a strength decrease was observed in heat curing mortar.

Many previous studies concluded that the nature of activator had significant effect on the alkali-activated slag properties, such as strength, microstructure, and shrinkage [5,15,27,28,29,30]. However, there are few publications in literature that have focused on the influence of activator on the AAS performance when subjected to elevated temperatures. Chi, M.C. [31] studied durability in high temperatures environment to 800 °C of AAS concrete while using alkaline activator with different concentrations of 4%, 5%, and 6% of Na_2_O by slag weight. The results exhibited high temperatures resistance of concrete was improved when increasing the Na_2_O concentration. Rashad, A.M. et al. [32] studied the effect of elevated temperatures on the AAS paste activated by Na_2_SO_4_ with concentrations 1%, 3% of Na_2_O equivalent by slag mass. The sample compressive strength was observed to increase slightly with an increase Na_2_O concentration after exposure to temperatures from 600 to 800 °C. Properties alteration of AAS paste with different sodium silicate concentration of 3.5%, 5.5%, 6.5%, 10.5% Na_2_O by slag weight when exposed to high temperatures up to 1000 °C was also investigated by Rashad, A.M. et al. [33]. The study indicated that the paste strength before and after exposure increased as the concentration of Na_2_O increased. Nevertheless, until now there has not been any publication which studied the influence of activator with different alkaline concentrations on the behavior of AAS mortar upon exposure to temperatures ranging from 200 to 1200 °C. Furthermore, the tensile strength of AAS mortar that was exposed to high temperatures has not also been investigated in previous studies. Consequently, the main goal of this paper is to determine the various Na_2_O concentration alkaline activated slag mortar mechanical strength and microstructure alteration after exposure to temperatures up to 1200 °C. Mechanical strength of mortar comprise compressive strength, flexural strength, and tensile strength.

## 2. Materials and Methods

### 2.1. Material Characterization

**Figure 1:**
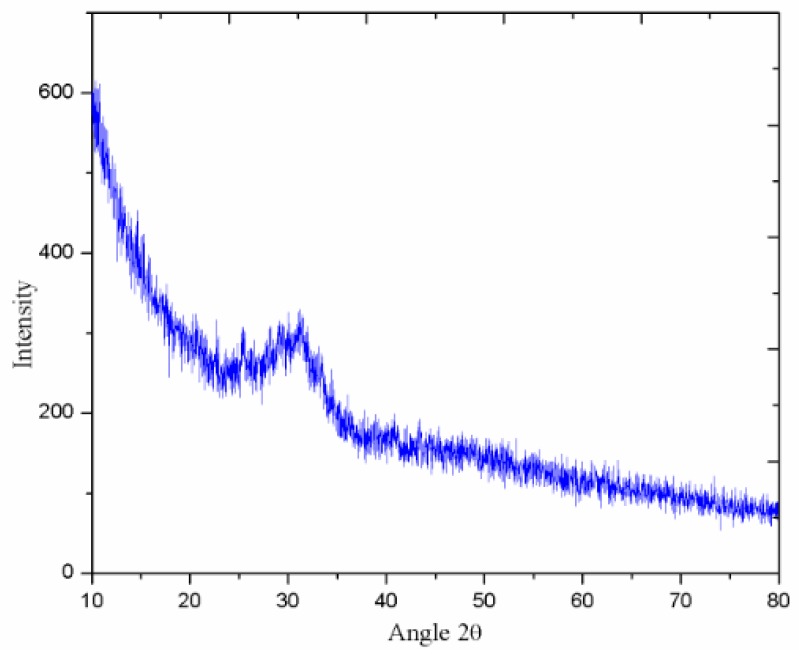
X-ray diffraction (XRD) pattern of blast furnace slag.

Blast furnace slag, which originated from South Korea, was used to synthesize the alkali activated slag mortar in current research. Slag has a specific surface area of 435 m^2^/kg (Blaine) and a density of 2.9 g/cm^3^. The activity index of slag at seven days and 28 days is 97% and 112%, respectively. The chemical composition and XRD analysis results of used slag material are presented in Table 1 and Figure 1. The XRD analysis diffractogram displays a wide diffusive hump between 25° and 35°, indicating that slag is mostly amorphous.

For material synthesis, blast furnace slag was activated by the alkaline activator, which was a combination of sodium silicate solution (water glass) and sodium hydroxide. Sodium silicate is liquid form with chemical composition comprising 26.4% Na_2_O, 8.2%SiO_2_, and 65.4% H_2_O by mass. Sodium hydroxide (NaOH) pellets was dissolved in sodium silicate solution to decrease the silica modulus to 1 and Na_2_O dosage of 4%, 6%, 8%, 10% by slag weight. The alkaline activator was prepared prior to mixing with slag 24 h. Local natural river sand (silica sand) with nominal maximum size of 4 mm and fineness modulus of 2.45 was used as fine aggregate to make mortar samples.

### 2.2. Mixture Proportion

The fine aggregate to slag mass ratio was 2.75. The alkaline activator portion was determined by dosage of Na_2_O per cent by slag weight. Four mortar mixtures with different activator concentration of 4%, 6%, 8%, and 10% Na_2_O by slag weight were named as A4, A6, A8, and A10, respectively. The water amount was adjusted to attain a water to solid ratio of 0.45 for all mortar mixtures. The solid portion included slag and solid component in alkaline activator.

### 2.3. Method

Blast furnace slag and fine aggregate were initially mixed in a 5 L capacity planetary blending machine for 1 min. Then, the mixture of alkaline activator and diluted water was poured into the machine bowl and continuously mixed for 2 min, followed by resting time of 1 min. During the resting period, the unmixed solids were scrapped from the sides and paddle into the mixing bowl. The whole mixture was mixed once again for 1 min. For casting, the fresh mortar mixture was poured into moulds of different shapes, depending on each mechanical property tests; 50 mm cube triplicate moulds for compressive strength test, 40 × 40 × 160 mm^3^ moulds for flexural strength test, and number “8” shaped moulds for tensile strength test. The specimens were then vibrated by using a vibrating table for 1 min to release any residual air bubbles. To prevent water evaporation, the specimens were covered with a thin plastic sheet and kept in the laboratory environment with a temperature of 20 ± 5 °C and humidity of 60 ± 5% for one day. The specimens were unmoulded and cured in the same condition for more 27 days prior to subject to high temperatures.

**Figure 2:**
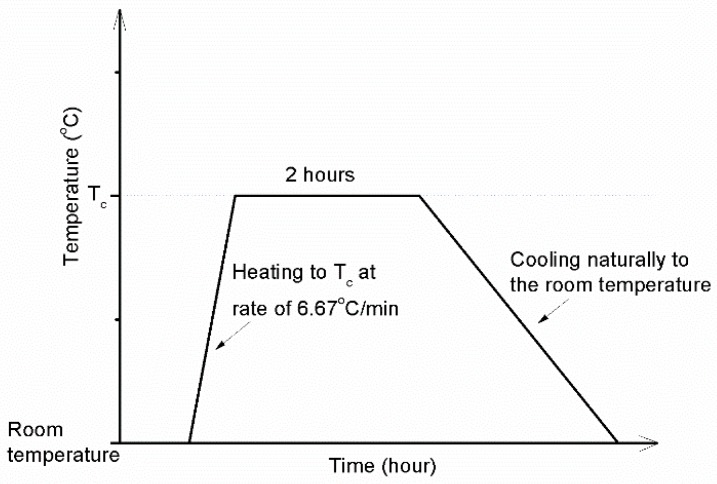
Temperature profile curve of furnace.

The mortar strengths were determined after curing period of 28 days according to standard ASTM C109 for compressive strength [34], ASTM C348 for flexural strength [35], and ASTM C190 for tensile strength [36]. The specimens, which were tested in the age of 28 curing days without exposure to elevated temperatures, were called the reference specimens or unexposed specimens. After 28 days of curing, the specimens were dried in oven at temperature of 105 ± 1 °C for 24 h prior to subject to high temperatures of 200 °C, 400 °C, 600 °C, 800 °C, 1000 °C, and 1200 °C (T_c_) by using an electrical heated furnace. For each temperature, the specimens were placed in the furnace and then heated at a rate of approximately 6.67 °C/min to obtain the determined temperature (T_c_). When reaching the target temperature (T_c_), the furnace temperature was maintained for 2 h. After that, the specimens were left in the furnace to cool naturally to ambient temperature. The furnace temperature versus time schedule is presented in Figure 2.

The exposed specimens were tested to determine the residual compressive strength, flexural strength, and tensile strength. After the compressive strength test, selected debris was immersed in acetone for three days to the stop hydration reaction. The debris was then filtered from acetone and dried in desiccator under vacuum. A part of dried samples was grounded and screened by using a 63 µm sieve. Fine particles passing a 63 µm sieve were used to analyze by X-ray diffraction (XRD) and thermogravimetric analysis (Thermogravimetric analysis (TGA)/Derivative thermogravimetric (DTG)) method. Nominated pieces were investigated by the scanning electron microscopy (SEM) with energy dispersive X-ray spectroscopy (EDS).

## 3. Results and Discussion

### 3.1. Compressive Strength

**Figure 3:**
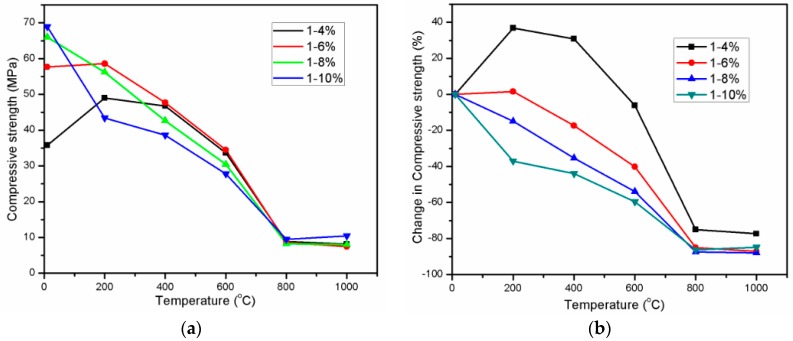
Residual compressive strength (**a**) and change in compressive strength (**b**) of alkali activated slag (AAS) mortar.

The behavior of mortar specimens with different Na_2_O concentrations when being exposed to elevated temperatures was examined by determining the residual mortar strength alteration, which is illustrated in Figure 3a. Figure 3b presents the change of exposed specimen strength in comparison with that of reference specimens. It can be seen from Figure 3 that the increase of Na_2_O concentration resulted in enhancement of the unexposed specimen compressive strength at 28 days. This result is consistent with previous studies [15,28]. With higher concentration of Na_2_O, the higher pH value of solution accelerated chemical reaction of alkali activation of slag, causing material strength gain. Increasing Na_2_O concentration from 4% to 6% led to great strength gain of approximately 61.2%. Nevertheless, the strength gain decreased gradually with further increasing concentration of Na_2_O above 6%. For instance, the A10 specimen strength was higher than that of A8 specimens by approximately 4.4%, whilst the A8 specimen strength increased 14.4% of A6 specimen strength. Parallel to the hydration process acceleration due to high alkaline activator, alkali activated slag exhibits high shrinkage deformation resulted from high sodium content [28,29] when the concentration of Na_2_O increases. This may explain for reduction in strength gain of AAS mortar when increasing Na_2_O concentration.

The residual compressive strengths of exposed specimens were highly dependent on Na_2_O concentration especially with the exposure temperature range from 200 to 400 °C. At 200 °C, the A4 specimens had a remarkable strength increase of 36.9% in comparison with strength of unexposed specimens. Increasing the exposure temperature up to 400 °C led to reduction in strength gain, but the residual strength at 400 °C was still higher than reference strength of specimens by 30.7%. This result is similar to study of Türker, H.T. et al. [26] about the ambient cured mortar sample. According to Türker, H.T. et al. [26], this strength enhancement was likely caused by heating effect accelerated the hydration process. However, the strength gain of A4 mortar specimen exposed to 200 and 400 °C was observed to decrease or be absent with further increase of Na_2_O concentration. Expose to 200 °C, the A6 specimens exhibited 1.6% enhancement of compressive strength whilst both A8 and A10 specimens possessed the great strength loss of 14.8% and 37%, respectively. It is noticeable that the loss of strength value of A10 mixture (37%) was equivalent to the strength gain value of A4 mixture (36.9%) at 200 °C. These results indicate that the enhancement in compressive strength at 200 °C decreases when increasing the concentration of Na_2_O. Influence of heating treatment on strength development of alkali-activated slag material was investigated in many previous studies [27,37,38]. Elevated temperature curing greatly accelerates strength gain in sodium silicate-activated slag material at early-age [37,39]. Furthermore, extension the length of time the samples were kept in ambient condition prior to heating treatment was observed to be unbeneficial for strength gain due to heating curing [26,37]. On the other hand, Gebregziabiher, B.S. et al. [39] found that the sample with elevated temperature curing exhibited the higher enhancement in strength as sodium oxide (Na_2_O) dosage increased. However, above results reveals that the strength gain due to later heating treatment occurs in sample that is activated by alkaline solution with low Na_2_O dosage.

With further exposing temperatures beyond 400 °C, A4 specimens exhibited slight strength reduction of 6.5% at 600 °C and considerable degradation in strength of 75.1% at 800 °C. The strength of A6 specimen gradually decreased to 17.3% and 40.2% at exposure temperature of 400 °C and 600 °C, respectively. The strength loss of both A8 and A10 specimens gradually alleviated when increasing temperature from 200 to 400 °C and 600 °C. It was noticeable from Figure 3 that all of the mortar mixtures lost significant strength and those strength values converged at the temperature of 800 °C. The slope of strength reduction line at temperature range from 400 to 600 and from 600 to 800 °C was observed to decrease when increasing the Na_2_O concentration from 4% to 10%. Throughout elevated temperature range from 200 to 600 °C, the A6 specimens exhibited the highest strength value among four mixtures specimens, whilst the lowest strength was observed in the mortar mixture with Na_2_O concentration of 10%.

**Figure 4:**
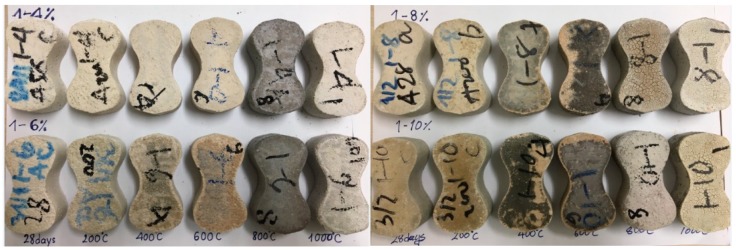
Photographs of AAS mortar samples activated with different concentration of Na_2_O before and after exposure to high temperatures from 200 to 1000 °C.

There was a minor change in compressive strength of all the mixtures at temperature range 800–1000 °C. In comparison with residual strength at 800 °C, A4, A6, and A8 specimen at 1000 °C exhibited the slight reduction in strength of 9%, 14.6%, and 3.6%, respectively, whilst A10 attained strength gain of 9.5%. This slight strength gain at temperature range 800–1000 °C of AAS mortar is similar to previous research [23,24], in which slag was activated by dried sodium silicate. With the highest Na_2_O concentration as 10%, the A10 specimens possessed the highest compressive strength at 800 and 1000 °C. It is obvious that the A4 specimens with the lowest Na_2_O concentration 4% attained the greatest strength increase and the lowest strength deterioration upon heating to high temperatures. During heating examination up to 1000 °C, there was no sign of spalling for all of the mixtures mortar, but a great amount of small cracks appeared on specimen surface at 800 °C and 1000 °C (Figure 4).

**Figure 5:**
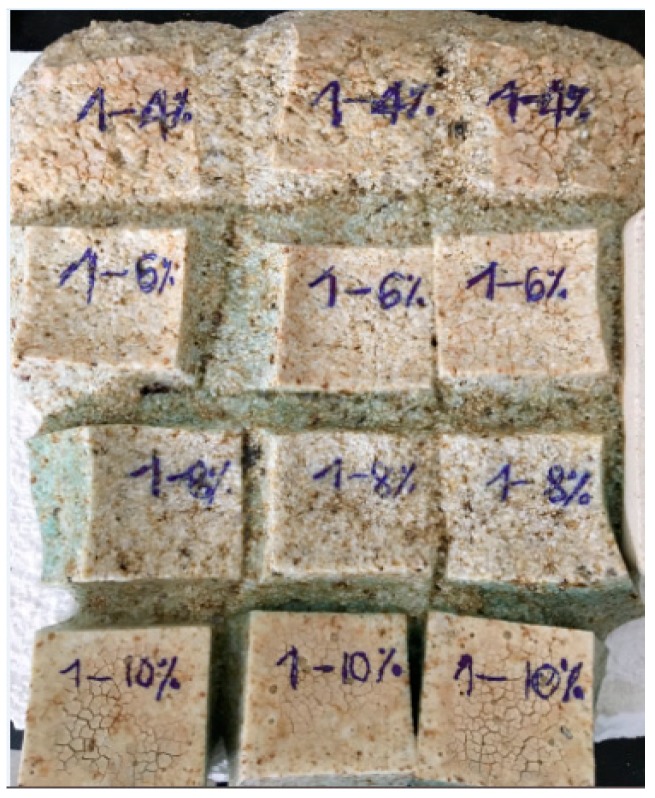
Photograph of AAS mortar samples after exposure to 1200 °C.

Raising the examination temperature up to 1200 °C resulted in deformation of all the mixture specimens due to melting phenomena. Therefore, the mechanical strength values could not be evaluated for samples after being exposed to 1200 °C. It is noticeable from Figure 5 that the deformed appearance was observed to be less in the specimens with higher concentration of Na_2_O.

According to previous studies [15,40,41], the brittleness of a material is evaluated by the ratio of flexural strength to compressive strength and the angle of internal friction. Angle of internal friction from Mohr envelope can be shown as ϕ = 0.5 × ARCTAN((CS − TS)/SQRT(CS × TS)), where CS and TS are the compressive and tensile strength. The brittleness of a material increases when ratio of flexural strength to compressive strength decreases and the angle of internal friction increases [40,41]. It can be seen from result in Table 2 that raising the Na_2_O concentration led to an increase in the brittleness property of AAS mortar in this study.

### 3.2. Flexural Strength

Flexural strength of AAS mortar in ambient temperature was highly dependent on Na_2_O concentration of activator. Increasing the Na_2_O concentration from 4% to 6% and 8% led to mortar strength gain of 30.9% and 36.8%, respectively. This result is consistent with finding of Duran Atiş, C. et al. [15]. However, the strength gain decreased slightly to 27.9% with a further increase in Na_2_O concentration to 10%. Flexural strength is more sensitive to micro-cracks than compressive strength [42]. The AAS mortar with higher Na_2_O concentration was observed to be more brittle, resulting in more micro-cracks due to higher shrinkage deformation [15]. Raising the concentration of Na_2_O led to not only the reaction acceleration but also the higher shrinkage deformation. This could explain the reason why there was the flexural strength reduction in mortar with Na_2_O concentration of 10%.

**Figure 6:**
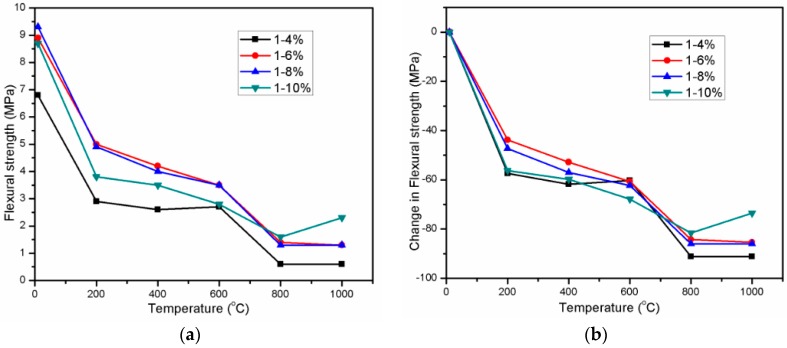
Residual flexural strength (**a**) and change in flexural strength (**b**) of AAS mortar.

Exposing mortars to 200 °C diminished the flexural strength of mortar significantly with all Na_2_O concentrations. More loss in strength was observed in samples with Na_2_O concentration of 4 and 10% than that of 6 and 8% Na_2_O samples. This flexural strength reduction could be attributed to further micro-crack formation resulted from shrinkage. As exposing to high temperature of 200 °C, ambient cured AAS mortar specimen experienced shrinkage which was combination of drying shrinkage due to evaporation of water from specimen and chemical shrinkage. According to Gu, Y.-M. et al. [42], the rapid reaction at relatively higher temperatures resulted in larger chemical shrinkage and induced micro-cracks in matrix at early ages, which developed with aging although the hardened paste got more compact. Increasing the exposing temperature from 200 to 400 °C and 600 °C alleviated the rate of reduction in flexural strength greatly. It is noticeable from Figure 6 that A4 specimen with the lowest Na_2_O concentration exhibited slight strength gain at exposing temperature range from 400 to 600 °C. The flexural strength deterioration increased when exposed to temperature from 600 to 800 °C. As seen in Figure 6, flexural strength behavior of AAS mortar in temperature range from 800 to 1000 °C was similar to that of mortar compressive strength. In comparison with residual strength of mortar at 800 °C, the A4, A6, and A8 specimens at 1000 °C exhibited no change or slight reduction in strength, whilst the slight strength increase occurred in A10 specimen. Throughout the exposed temperatures, the residual strength of A6 and A8 specimens was equivalent each other. The flexural strength variation trend of mortar was different to that in result of some previous studies [23,24]. According to Zuda, L. et al. [23], there was no chance in flexural strength of AAS mortar, which used quartz sand as fine aggregate when exposing to 200 °C. When using electrical porcelain as fine aggregate of AAS mortar, Zuda, L. et al. [24] found that mortar flexural strength altered slightly at temperature range from 200 to 1000 °C.

### 3.3. Tensile Strength

**Figure 7:**
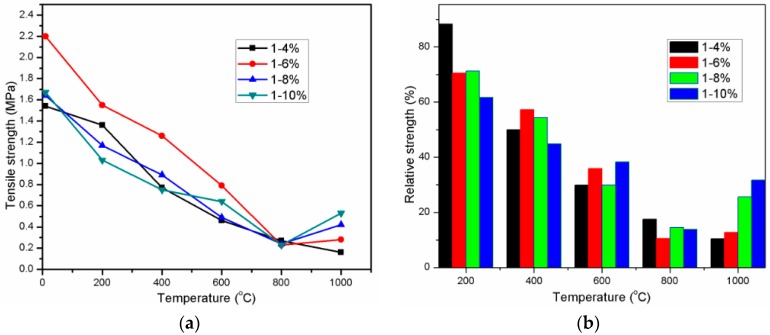
Residual tensile strength (**a**) and relative tensile strength (**b**) of AAS mortar.

The tensile strength which was determined by testing “8” shaped mortar specimens at ambient temperature and after exposure to high temperatures is given in Figure 7a. Figure 7b presents the relative tensile strength of mortar that is exposed to high temperatures in comparison with unexposed mortar strength. Similar to compressive and flexural strength, the Na_2_O concentration of activator has an important role in tensile strength of unexposed specimens. The AAS mortar using activator with Na_2_O concentration of 6% possessed the highest tensile strength value, which was approximately 1.43, 1.34, and 1.32 times higher than that of A4, A8, and A10 specimen, respectively. Raising Na_2_O concentration in activator to 6% led to grow of the tensile strength value, however the strength was investigated to decrease with further increase of Na_2_O concentration beyond 6%. According to Duran Atiş, C. et al. [15], there is direct correlation between the tensile strength and the brittleness of AAS mortar. The mortar with higher brittleness is higher shrinkage, and thus, the tensile strength is lower. The mortar is more brittle, the tensile strength is lower due to cracking by shrinkage deformation resulted from high Na concentration. When exposed to high temperatures from 200 to 800 °C, the tensile strength of all mixture AAS mortar decreased rapidly and then converged at temperature of 800 °C. The tensile strength of AAS mortar is strongly dependent on the paste-aggregate bond strength [43]. As exposing to high temperatures, the thermal incompatibility between AAS paste and fine aggregate resulted in weakening the paste-aggregate bond. In addition to weak AAS paste-aggregate bond, the development of pre-existing micro-cracks as well as new crack formation due to thermal shrinkage also caused deterioration in tensile strength of mortar. Throughout high temperature range from 200 to 600 °C, the A6 mortar specimen still possessed the highest residual strength in 4 mortar mixtures.

It can be seen in Figure 7 that Na_2_O concentration has apparent influence on residual tensile strength of mortar at temperature of 1000 °C. In comparison with strength of mortar at 800 °C, the strength of A6, A8, and A10 mortar specimens at 1000 °C was observed to increase slightly, whilst strength loss occurred in the A4 mortar. The AAS mortar with higher Na_2_O concentration exhibited higher tensile strength enhancement. For instant, the residual strength of A10 specimen after exposed to 1000 °C was 2.3 times higher than that of mortar at 800 °C. This ratio decreased to 1.8, 1.2, and 0.6 for mortar with Na_2_O concentration of 8%, 6%, and 4%, respectively.

**Figure 8:**
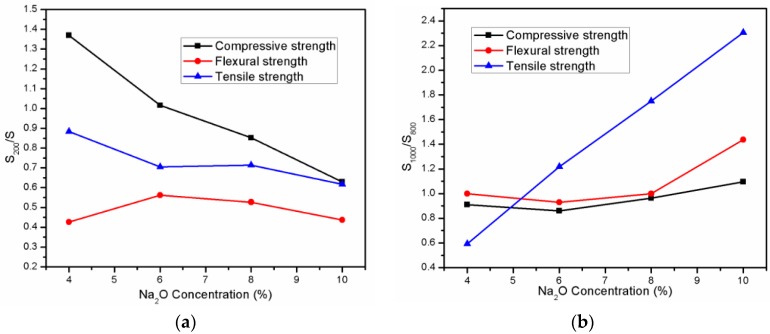
Relation between Na_2_O concentration and the strength of AAS mortar after exposure to high temperature range: (**a**) From laboratory temperature to 200 °C; and, (**b**) From 800 to 1000 °C.

According to above results, there was clear correlation between the brittleness property and residual mechanical strength of AAS mortar exposed to high temperature range from laboratory temperature to 200 °C and from 800 to 1000 °C. Relation between Na_2_O concentration and the mechanical strength variation in temperature range from ambient to 200 °C and from 800 to 1000 °C is, respectively, shown in Figure 8a,b, respectively. After exposed to 200 °C, the loss in compressive strength was higher in AAS mortar with higher brittleness. In contrast, the mechanical strength of the AAS mortar had a tendency to increase with increasing the brittleness at temperature range 800–1000 °C.

### 3.4. Thermogravimetric Analysis

**Figure 9:**
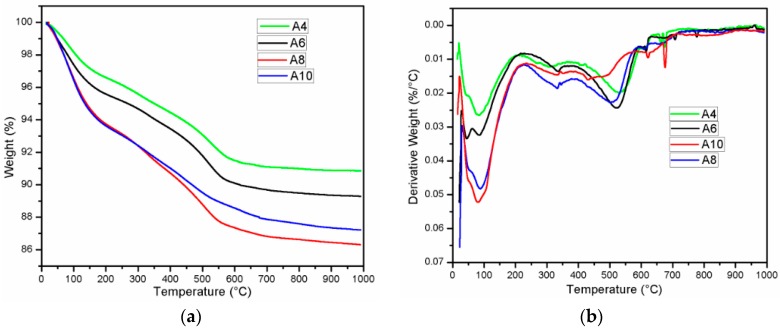
Thermogravimetric analysis (TGA) analysis (**a**) and Derivative thermogravimetric (DTG) analysis result (**b**) for AAS mortar at 28 days.

Figure 9 presents the result of Thermogravimetric analysis (TGA) and Derivative thermogravimetric (DTG) of mortar with different Na_2_O concentration of 4%, 6%, 8%, and 10%. For four samples, the main DTG peak centered at approximately 90 °C is result of dehydration of calcium silicate hydrate (C-S-H) [44]. It is noted from Figure 9b that the DTG peak of C-S-H is larger and sharper when increasing the Na_2_O concentration in mortar. It reveals that more hydration product C-S-H is formed in AAS mortar with a higher concentration of Na_2_O. This is consistent with the sharp mass loss of A10 and A8 mortar sample due to release of free water and OH groups from matrix [26] before around 120 °C (TGA curve in Figure 9a). The rapid migration and evaporation of water resulted in more micro-cracks in the mortar structure. This also may explained the reason why the significant deterioration in compressive strength occurred in A8 and A10 mortar when exposed to 200 °C. The reduction in mass loss was observed in all mixture specimens for temperature higher 150 °C. On the other hand, the wide DTG peak at approximately 550 °C due to decomposition of calcite (CaCO_3_) [45] was apparently observed to be present in A4, A6, and A8 samples. These DTG peaks are consistent with sharp reduction in mass of A4, A6, and A8 sample in temperature range from 450 to 560 °C in TGA curve. Furthermore, the slope of mass reduction line in this temperature range increased with an increase of Na_2_O concentration. The significant degradation in mass reduction occurred abruptly at approximately temperature of 560 °C. Beyond this temperature, there was minor change in the percentage of residual mass and all of the samples possessed the minimum mass value at 1000 °C. It can be seen from Figure 9a that the elevated temperatures treatment caused the highest mass loss in mortar with Na_2_O concentration of 8%, followed with Na_2_O concentration of 10%, 6%, and 4%, respectively.

### 3.5. XRD Analysis

**Figure 10:**
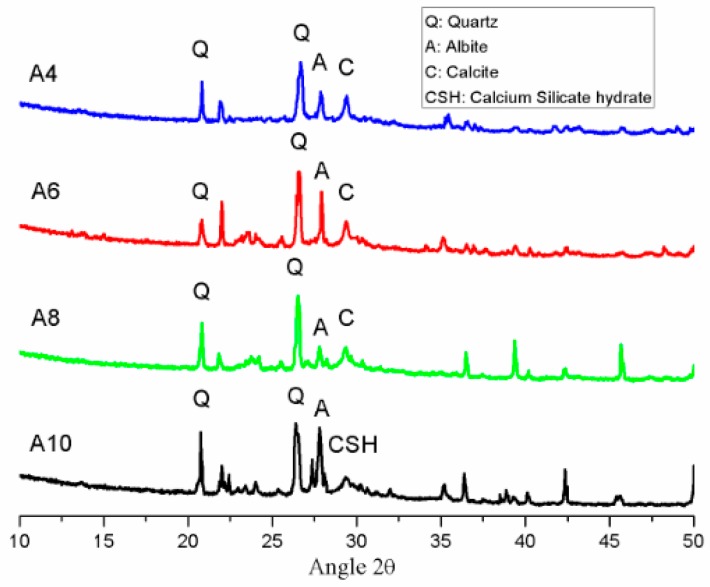
XRD patterns of AAS mortar with different concentration of Na_2_O before exposure.

Figure 10 displays the XRD traces of unexposed AAS mortar with different Na_2_O concentrations after 28 days of curing in ambient condition. The predominant crystalline phase of Quartz (SiO_2_) was detected with minor reflections of Albite (NaAlSi_3_O_8_), Calcite (CaCO_3_), and calcium silicate hydrate (CaO·SiO_2_·nH_2_O) (C-S-H). From previous studies [33,45], the peak of C-S-H was present in overlapping with trace of Calcite (CaCO_3_). The XRD trace of Calcite (CaCO_3_) was identified clearly in A4, A6, and A8 mortar samples. However, Calcite reflection was not found in mortar specimen with Na_2_O concentration of 10%. This XRD result is consistent with DTA and TGA analysis.

### 3.6. Microstructural Analysis

**Figure 11:**
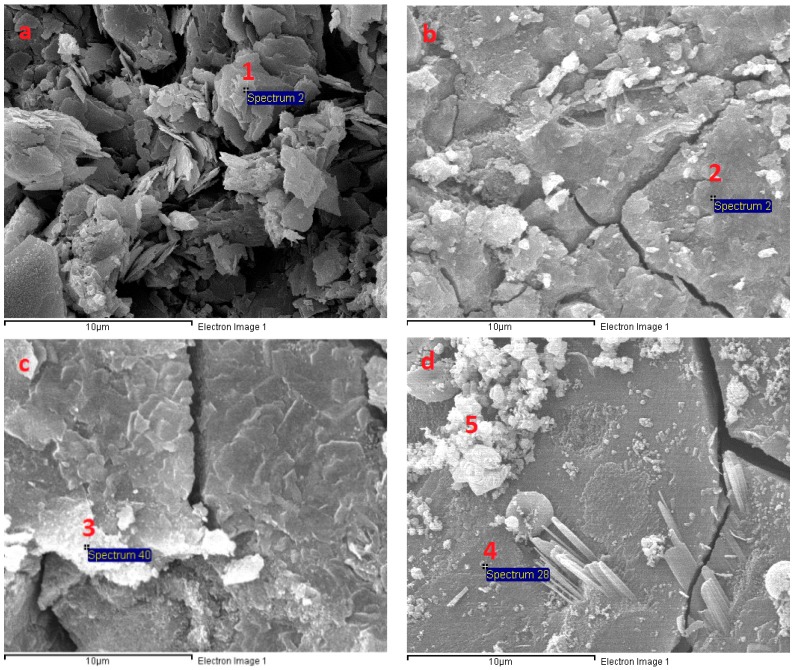
Scanning electron microscopy (SEM) micrograph of fracture surface of AAS mortar at 28 days: (**a**) A4; (**b**) A6; (**c**) A8; and, (**d**) A10.

**Figure 12:**
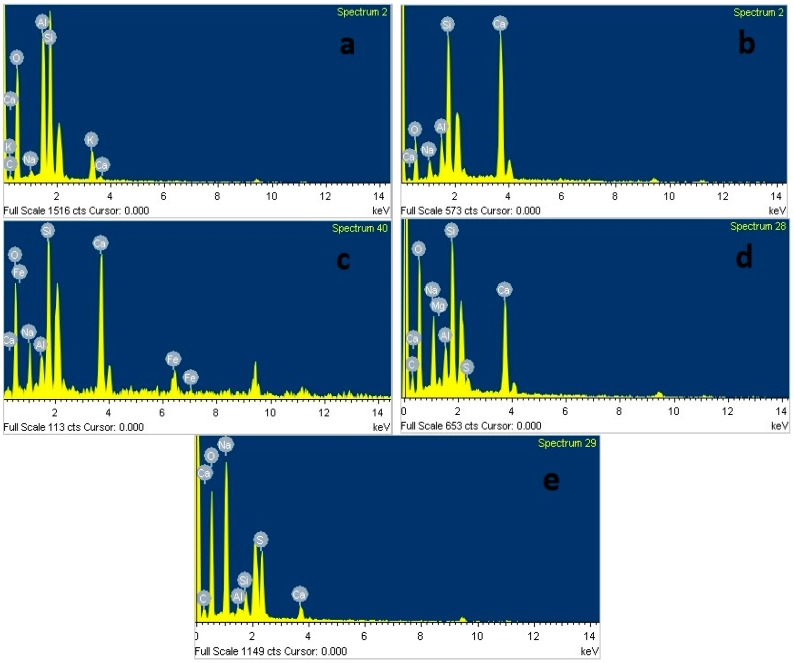
Energy dispersive X-ray spectroscopy (EDS) image of spots in Figure 11: (**a**) Spot labeled 1; (**b**) Spot labeled 2; (**c**) Spot labeled 3; (**d**) Spot labeled 4; and, (**e**) Spot labeled 5.

The SEM micrograph in Figure 11 shows the microstructure of AAS mortar activated by alkaline solution with different Na_2_O concentrations at the age of 28 days curing in ambient condition. It is noted from Figure 11 that the AAS mortar is highly dependent on concentration of Na_2_O. The sample with concentration of 4% Na_2_O exhibited a porous microstructure and unhydrated slag particles. Figure 12a presents the EDS trace of spot marked 1 on A4 specimen surface (Figure 11) where the dominant elements were detected to be Si and Al, whilst Ca element was present with minor amount. The Calcium amount is not enough to make a reaction with Silicate component for C-S-H formation. These results prove that the low activation process of slag was resulted from low alkaline activator with Na_2_O concentration of 4%. As seen in Figure 11b–d, the structure became denser and well-packed with increasing the concentration of Na_2_O. Specimens A6, A8, and A10 display micro-cracks due to high shrinkage deformation. On the other hand, the EDS spectra in Figure 12b–d reveals that Ca element amount at spots marked 2, 3, 4 in Figure 11b–d increased significantly to be dominant with Si element when increasing concentration of Na_2_O. Furthermore, EDS spectra (Figure 12e) of spot 5 at Figure 11d indicates that there was free sodium component in A10 mortar sample. Furthermore, Sodium substituted calcium silicate hydrate (N-C-S-H) with low Ca/Si is known as the main reaction product of alkali activated slag [46,47]. Malolepszy, J. [48] postulated the formation of a solid solution of Na_2_O-CaO-SiO_2_-H_2_O (N-C-S-H), since Na^+^ ions in alkali-activated cement have a very low solubility in water. It is well known that the Ca/Si ratio has a significant influence on properties of C-S-H. EDS image in Figure 12 reveals that Ca/Si ratio decreased with raising Na_2_O concentration from 6% to 10%. The Ca/Si ratio reduction causes an improvement in binding ability of C-S-H [46,49,50], led to a compressive strength gain of AAS mortar.

**Figure 13:**
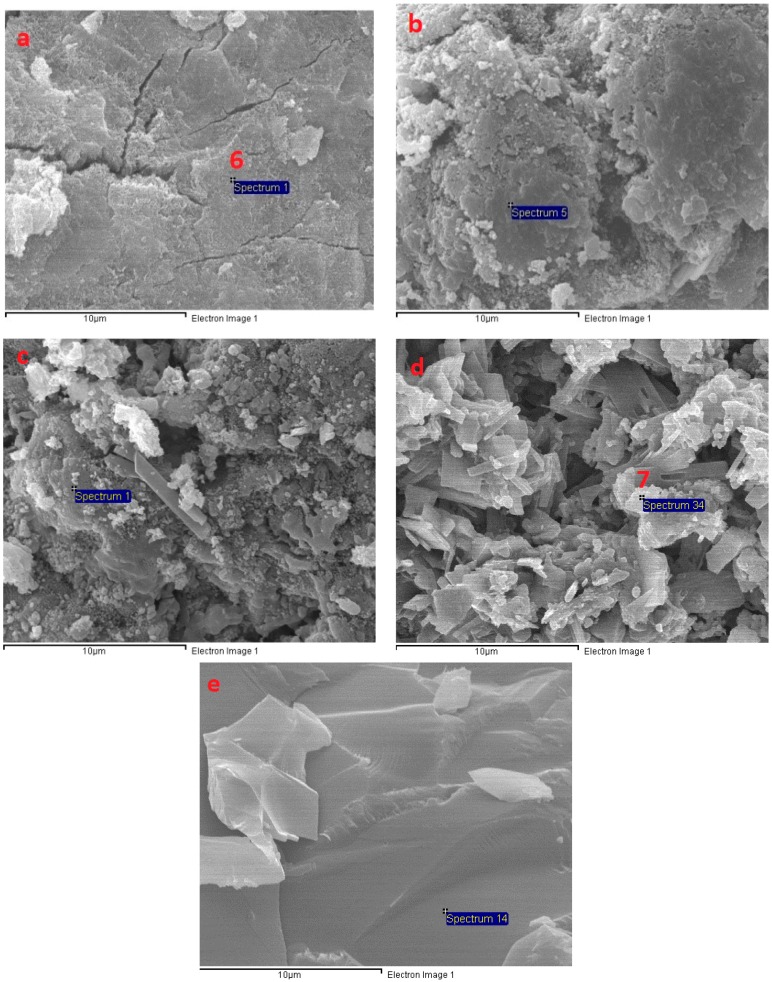
SEM micrographs of fracture surface of A4 at different temperatures: (**a**) 200 °C; (**b**) 600 °C; (**c**) 800 °C; (**d**) 1000 °C; and, (**e**) 1200 °C.

**Figure 14:**
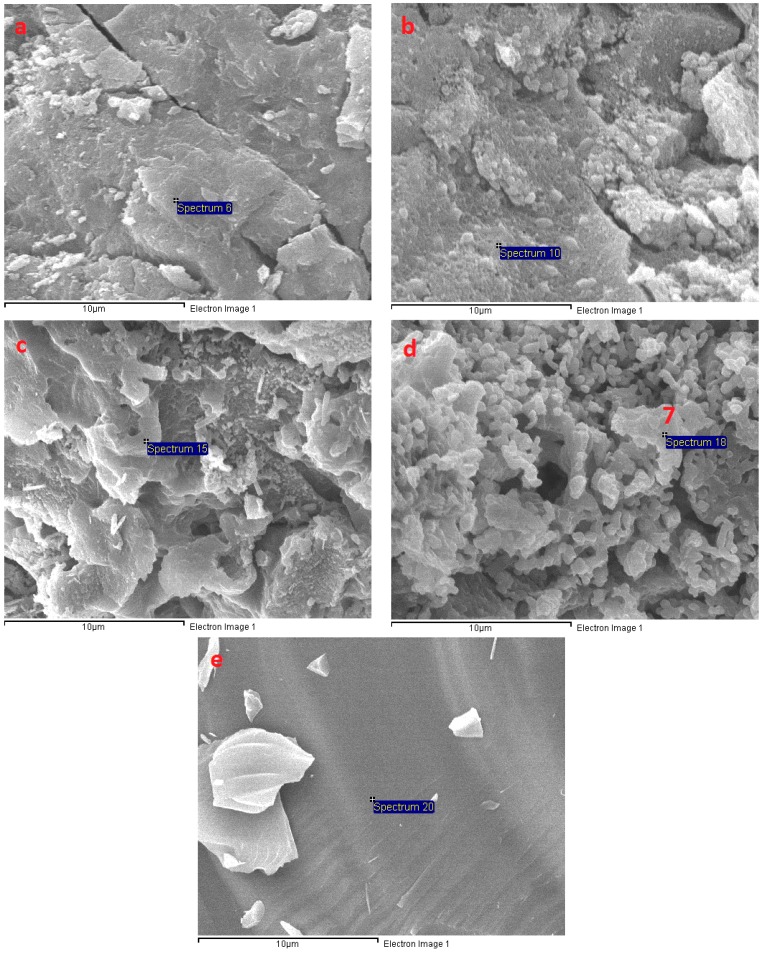
SEM micrographs of fracture surface of A6 at different temperatures: (**a**) 200 °C; (**b**) 600 °C; (**c**) 800 °C; (**d**) 1000 °C; and, (**e**) 1200 °C.

**Figure 15:**
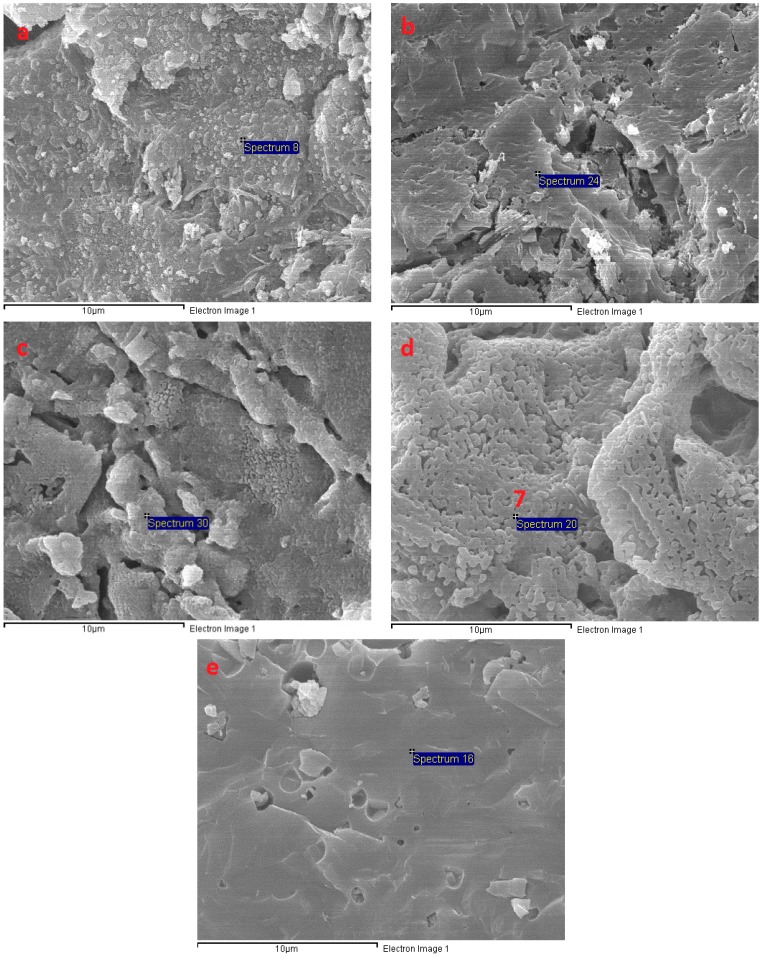
SEM micrographs of fracture surface of A10 at different temperatures: (**a**) 200 °C; (**b**) 600 °C; (**c**) 800 °C; (**d**) 1000 °C; and, (**e**) 1200 °C.

**Figure 16:**
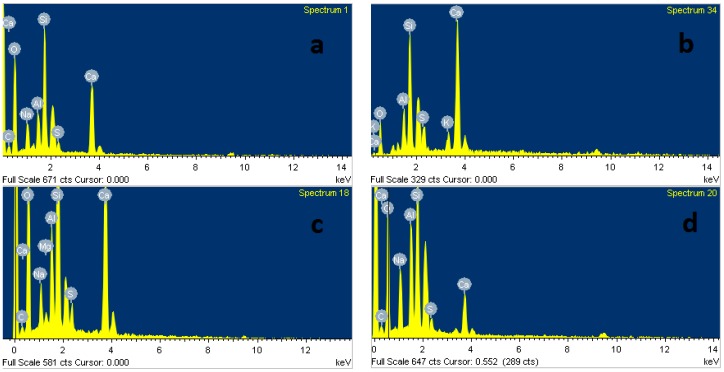
EDS image of: (**a**) Spot labeled 6 in Figure 13; (**b**) Spot labeled 7 in Figure 13; (**c**) Spot labeled 7 in Figure 14; and, (**d**) Spot labeled 7 in Figure 15.

The microstructure transformation of AAS mortar after exposure to elevated temperatures up to 1200 °C is given in Figure 13, Figure 14 and Figure 15. It is noticeable that, as samples exposed to 200 °C, the porous structure of unexposed mortar with the lowest Na_2_O concentration of 4% transformed to be compact structure with few hair-line micro-cracks. This change could be attributed to more hydration product resulted from the reaction acceleration due to the heating effect. Furthermore, migration of water before 200 °C occurred more easily from the porous structure of A4 mortar. This explains for the compressive strength gain of A4 mixture mortar at 200 °C. Moreover, Ca element amount at A4 fragment surface was investigated to increase highly from EDS spectra (Figure 16a) of spot marked 6 in Figure 13, revealing the formation of C-S-H. As seen in Figure 14, there was no significant change in microstructure of A6 mixture mortar at 200 °C. Contrary to A4 sample, the rapid migration and escape of a large amount of water from unexposed dense structure of A8 and A10 specimens caused the significant degradation in strength after exposure to 200 °C. This explains the reason why the microstructure of mortar with Na_2_O concentration 10% possessed more cracks and rough surface at 200 °C (Figure 15). Micro-cracks were observed to be present on mortar surface (Figure 13, Figure 14 and Figure 15) led to the drop of flexural and tensile strength when exposed to high temperatures.

One of the main reasons for the deterioration in strength of AAS mortar at high temperature was thermal incompatibility between AAS matrix and fine aggregate. For exposure to elevated temperature, the AAS paste contraction occurred by the loss of water, whilst fine aggregate expands, resulting in weakening the bond between paste and aggregate. In addition, the occurrence of calcite decomposition process in mortars with Na_2_O concentration of 4%, 6%, and 8% at a temperature of around 550 °C weakened mortar structure. This likely to explain reason why A10 specimen exhibited the lower deterioration rate in strength at temperature range from 400 to 600 °C. Furthermore, the great strength reduction result of AAS mortar from 600 to 800 °C is consistent with the significant damaged structure of mortar, which is presented in Figure 13, Figure 14 and Figure 15.

Increasing the exposing temperature from 800 to 1000 °C resulted in the significant alteration of AAS mortar structure due to sintering process, a significant increase in porosity and no sign of cracks was observed. This remarkable microstructure alteration may be the cause for slight change in mechanical strength of mortar from 800 to 1000 °C. As seen in Figure 13, Figure 14 and Figure 15, alkali activated mortar structure at 1000 °C was denser with an increasing concentration of Na_2_O. Figure 16 presents the EDS spectra of spots marked 7 in Figure 13, Figure 14 and Figure 15, revealing a reduction in Ca/Si ratio when raising Na_2_O concentration from 4% to 10%. The reduction of Ca/Si ratio in C-S-H structure indicates an enhancement in binding ability of C-S-H [49,50]. Both SEM and EDS result could explain for the higher strength of A10 mortar sample when exposing to 1000 °C. As sample exposed to temperature of 1200 °C, previous porous microstructure of all mortar mixtures was transformed to a considerable dense structure with smooth surface. The cracks and pores in structure at previous temperature were healed and filled by melting of AAS when exposed to 1200 °C. In spite of having highly dense structure, AAS mortar specimens observed to be deformed due to melting at 1200 °C and the intensity of deformation was higher in mortar with lower Na_2_O concentration. This result could be attributed to binding ability of C-S-H in mortar structure, which was improved when increasing concentration of Na_2_O.

## 4. Conclusions

Based on above experimental results and discussion, the study reveals the following conclusions:

The Na_2_O concentration of alkaline activator has a great influence on mechanical strength of unexposed alkali-activated slag mortar, higher compressive strength with higher concentration of Na_2_O. Moreover, raising the concentration of Na_2_O led to increasing the brittleness of AAS mortar.

The compressive strength gain at 200 °C was observed in AAS mortar with a low Na_2_O concentration of 4 and 6%, whilst mortar activated with Na_2_O concentration of 8 and 10% exhibited a great reduction in strength.

The variation in residual flexural strength of AAS mortar was similar to tensile strength when exposed to high temperatures below 800 °C. The AAS mortar with Na_2_O concentration of 6% exhibited the highest mechanical strength with exposing temperature below 800 °C.

The difference in residual mechanical strength of all mixture mortar was negligible at the exposure temperature range from 800 to 1000 °C. The mechanical strength of mortar at 1000 °C had a tendency to increase with higher Na_2_O concentration and brittleness.

The highest mass loss after exposure was observed in alkali activated mortar with Na_2_O concentration of 8%. Calcite (CaCO_3_) was not found in alkali activated slag mortar with Na_2_O concentration of 10%.

All mixture AAS mortar deformed significantly due to melting phenomena after being exposed to 1200 °C. The deformation was observed to be less with increasing concentration of Na_2_O from 4% to 10%. The microstructure of mortar was changed to be highly dense with smooth surface when exposed to 1200 °C irrespective of Na_2_O concentration.

## Figures and Tables

**Table 1:** Chemical composition of used blast furnace slag.

Oxide	SiO_2_	CaO	Al_2_O_3_	Fe_2_O_3_	MgO	SO_3_	Na_2_O	K_2_O	LOI
(%)	33.81	41.24	15.19	0.41	5.54	2.51	0.25	0.61	0.18

**Table 2:** Mechanical strength, ratio of flexural strength to compressive strength (FS/CS) and internal friction angle (ϕ) of AAS mortar at 28 days.

Mixture	Compressive Strength CS (MPa)	Flexural Strength FS (MPa)	Tensile Strength TS (MPa)	FS/CS	ϕ (Radian)
A4	35.8	6.8	1.54	0.18994	0.67868
A6	57.7	8.9	2.2	0.15425	0.68526
A8	66	9.3	1.64	0.14091	0.70527
A10	68.9	8.7	1.67	0.12627	0.70629
